# Impaired Ganglion Cell Function Objectively Assessed by the Photopic Negative Response in Affected and Asymptomatic Members From Brazilian Families With Leber's Hereditary Optic Neuropathy

**DOI:** 10.3389/fneur.2020.628014

**Published:** 2021-01-18

**Authors:** Gabriel Izan Santos Botelho, Solange Rios Salomão, Célia Harumi Tengan, Rustum Karanjia, Felipo Victor Moura, Daniel Martins Rocha, Paula Baptista Eliseo da Silva, Arthur Gustavo Fernandes, Sung Eun Song Watanabe, Paula Yuri Sacai, Rubens Belfort, Valerio Carelli, Alfredo Arrigo Sadun, Adriana Berezovsky

**Affiliations:** ^1^Departamento de Oftalmologia e Ciências Visuais, Escola Paulista de Medicina, Universidade Federal de São Paulo, São Paulo, Brazil; ^2^Departamento de Neurologia e Neurocirurgia, Escola Paulista de Medicina, Universidade Federal de São Paulo, São Paulo, Brazil; ^3^Doheny Eye Institute, University of California Los Angeles, Los Angeles, CA, United States; ^4^Department of Ophthalmology, Doheny Eye Center, David Geffen School of Medicine at UCLA, Los Angeles, CA, United States; ^5^Ottawa Eye Institute, University of Ottawa, Ottawa, ON, Canada; ^6^Ottawa Hospital Research Institute, Ottawa, ON, Canada; ^7^Instituto da Visão-IPEPO, São Paulo, Brazil; ^8^Department of Biomedical and NeuroMotor Sciences (DIBINEM), University of Bologna School of Medicine, Bologna, Italy

**Keywords:** leber's hereditary optic neuropathy, photopic negative response, retinal ganglion cell, visual evoked cortical potentials, electroretinography

## Abstract

**Purpose:** The photopic negative response (PhNR) is an electrophysiological method that provides retinal ganglion cell function assessment using full-field stimulation that does not require clear optics or refractive correction. The purpose of this study was to assess ganglion cell function by PhNR in affected and asymptomatic carriers from Brazilian families with LHON.

**Methods:** Individuals either under suspicion or previously diagnosed with LHON and their family members were invited to participate in this cross-sectional study. Screening for the most frequent LHON mtDNA mutations was performed. Visual acuity, color discrimination, visual fields, pattern-reversal visual evoked potentials (PRVEP), full-field electroretinography and PhNR were tested. A control group of healthy subjects was included. Full-field ERG PhNR were recorded using red (640 nm) flashes at 1 cd.s/m^2^, on blue (470 nm) rod saturating background. PhNR amplitude (μV) was measured using baseline-to-trough (BT). Optical coherence tomography scans of both the retinal nerve fiber layer (RNFL) and ganglion cell complex (GCC) were measured. PhNR amplitudes among affected, carriers and controls were compared by Kruskal-Wallis test followed by *post-hoc* Dunn test. The associations between PhNR amplitude and OCT parameters were analyzed by Spearman rank correlation.

**Results:** Participants were 24 LHON affected patients (23 males, mean age=30.5 ± 11.4 yrs) from 19 families with the following genotype: m.11778G>A [*N* = 15 (62%), 14 males]; m.14484T>C [*N* = 5 (21%), all males] and m.3460G>A [*N* = 4 (17%), all males] and 14 carriers [13 females, mean age: 43.2 ± 13.3 yrs; m.11778G>A (*N* = 11); m.3460G>A (*N* = 2) and m.14484T>C (*N* = 1)]. Controls were eight females and seven males (mean age: 32.6 ± 11.5 yrs). PhNR amplitudes were significantly reduced (*p* = 0.0001) in LHON affected (−5.96 ± 3.37 μV) compared to carriers (−16.53 ± 3.40 μV) and controls (−23.91 ± 4.83; *p* < 0.0001) and in carriers compared to controls (*p* = 0.01). A significant negative correlation was found between PhNR amplitude and total macular ganglion cell thickness (*r* = −0.62, *p* < 0.05). Severe abnormalities in color discrimination, visual fields and PRVEPs were found in affected and subclinical abnormalities in carriers.

**Conclusions:** In this cohort of Brazilian families with LHON the photopic negative response was severely reduced in affected patients and mildly reduced in asymptomatic carriers suggesting possible subclinical abnormalities in the latter. These findings were similar among pathogenic mutations.

## Introduction

Leber's hereditary optic neuropathy (LHON) is a disease characterized by a sub-acute, painless loss of central vision, either simultaneously or in one eye followed by the other eye within weeks to months, affecting mainly young male adults between 15 and 35 years of age (1). The loss of vision is due to selective vulnerability of retinal ganglion cells (RGCs) in the papillomacular bundle that causes central scotoma and subsequent optic atrophy (2, 3).

The disease is caused by mutations in the mitochondrial DNA (mtDNA) that disrupt critical complex I subunits of the mitochondrial respiratory chain, causing impaired cellular ATP synthesis and increased production of reactive oxygen species (4, 5). The main mutations are m.11778G>A (ND4), m.14484T>C (ND6) and m.3460G>A (ND1) considered the three primary variations and representing over 90% of all LHON cases (5).

LHON is the most common of the mtDNA diseases, but epidemiological studies on prevalence and incidence involving different countries are scarce. A recent meta-analysis in Europe, estimated LHON prevalence of one in 40,000 (6). LHON is more frequent in males with the male/female ratio varying from 3:1 to 8:1, depending on the LHON mutation and the population studied (1, 7). The penetrance of the disease is incomplete with only about 50% of males and 10% of females carrying a genetic defect becoming affected and a substantial number of individuals along the maternal line carrying the genetic defect remaining asymptomatic lifelong (1).

A very large Brazilian pedigree with m.11778G>A/haplogroup J LHON (SOA-BR) has been extensively studied (3, 8–32) but information on other Brazilian LHON families has not been thoroughly investigated. A major obstacle in a developing country is the poor access to genetic analysis which provides confirmation of one of the three primary LHON mtDNA mutations, even though a strong clinical suspicion of the disease was present based on symptoms and neuro-ophthalmological assessment (33).

The involvement of RGCs on the LHON pathophysiology has been confirmed by fundoscopy, optical coherence tomography (OCT) and histopathological studies (23, 33–35). Recently, it was discovered that RGCs also generate a slow negative wave response observable on the full-field electroretinogram (ff-ERG) immediately following the b-wave of the cone response. This component of the ERG is referred to as the photopic negative response (PhNR) (36) and it has not been fully incorporated in conventional full-field ERG protocols as it is recommended as an expanded testing protocol by the ISCEV (37, 38). Reduced PhNR amplitudes have been reported in patients with RGCs pathologies such as glaucoma (39–42), optic atrophy (43, 44), childhood optic glioma (45), retinal vascular diseases (46–50) and idiopathic intracranial hypertension (51, 52).

A previous study including only members from the SOA-BR pedigree reported that PhNR amplitude is significantly decreased in patients affected by LHON compared to carriers and there was also a decrease in PhNR in carriers, suggesting potential subclinical RGC dysfunction (32). Electrophysiological assessment including PhNR performed in LHON families from the United Kingdom harboring one of the three common mtDNA mutations, was attenuated in affected individuals (53).

Our purpose was to prospectively investigate a cohort of Brazilian families other than the extensively studied SOA-BR pedigree, aiming to assess ganglion cell function by PhNR in affected and asymptomatic carriers. Additionally, other clinical features were studied by comprehensive ophthalmic and electrophysiological testing including visual acuity, fundus exam, optical coherence tomography, color discrimination, visual fields, visually evoked potentials and full-field electroretinography.

## Methods

In this prospective, observational, cross-sectional study, patients with a clinical suspicion or diagnosis of LHON and their family members were invited for a free-of-charge assessment in the Clinical Electrophysiology of Vision Laboratory of the Federal University of São Paulo (UNIFESP) from August 2018 to January 2020. The inclusion criteria were the presence of the following features: (a) clinical symptoms suggesting LHON including painless and subacute blurred vision either bilateral or in one eye followed by the other; (b) vascular tortuosity of the central retinal vessels, swelling of the retinal nerve fiber layer and peripapillary telangiectatic microangiopathy and optic disc atrophy or paleness; (c) dyschromatopsia or color blindness and central scotoma, and (d) family history of individuals with bilateral sequential visual loss in the maternal line. Subjects with macular degeneration or signs of pathology of the optic nerve other than LHON were excluded.

A control group was included with healthy volunteers recruited among students and employees from the Federal University of São Paulo. Inclusion criteria for the control group were: visual acuity with current correction in either eye = 0.0 logMAR and normal ophthalmic examination. The exclusion criteria were: high ametropia (spherical equivalent = ±5.00 diopters), any systemic disease, family history of glaucoma, history of previous eye surgery and history of hereditary eye diseases.

This study has been approved by the Committee of Ethics in Research of the Federal University of São Paulo and adhered to the tenets of the Declaration of Helsinki. All participants provided informed consent.

## Procedures

### Clinical Parameters

A thorough history was taken to determine demographic features as age, sex, associated symptoms, age at onset of vision loss and time between the first and second affected eyes. Family history of LHON was collected and a family pedigree was elaborated. Any known exposure to environmental toxins, tobacco, alcohol, and drugs was also noted. Additional information included whether patients were currently being treated with idebenone.

### Visual Electrophysiological Assessment

#### Pattern-Reversal Visually Evoked Potential and Flash Visually Evoked Potential

PRVEP and FVEP were done with natural pupils in a darkened room using the UTAS E-3000 Electrodiagnostic System (LKC Technologies Inc., Gaithersburg, MD, USA), in accordance with International Society of Clinical Electrophysiology of Vision (ISCEV) guidelines (54). PRVEP of each eye were obtained using electrodes placed according to the 10–20 system. The active, reference, and ground electrodes were placed at O_z_, FP_z_, and C_z_ respectively. Stimuli were presented in a monochromatic CRT display at a 1 m distance using two check sizes subtending 15' and 60' visual angles.

The PRVEP waveforms were triphasic. The main positive deflection was the P100, the preceding and following negative deflections were the N75 and the N135, respectively. Peak-to-peak amplitudes were measured from the first negative deflection (N75) to the following positive deflection (P100) and expressed in microvolts (μV). Peak times were measured for P100 in milliseconds (ms). Amplitudes were classified as normal or reduced and P100 peak times as normal or delayed in relation to normative cutoffs obtained from normal values of our own laboratory (55).

FVEP was presented inside a Ganzfeld dome and the waveforms were composed by successive deflections and named in order of appearance. The first and the second positive deflections were named P1 and P2, respectively, and their preceding negative deflections, N1 and N2. Peak-to-peak amplitudes (μV) were measured for N1–P1 and N2–P2 complexes. Peak times (ms) were measured for all deflections (N1, P1, N2, and P2).

#### Full-field ERG

ERGs were performed following ISCEV standardized protocol in both eyes (37). Both pupils were dilated (pupil diameter >7 mm) after administering a drop of tropicamide 1% and a drop of phenylephrine 10%, and all subjects were dark-adapted for 30 min. The corneal surface was anesthetized with two drops of tetracaine 1.0% and a bipolar contact lens electrode (Burian-Allen bipolar electrode, Hansen Ophthalmic Development Lab, Coralville, IA, USA) was placed on the corneal surface with a drop of methylcellulose 2%. A gold cup ground electrode was applied to the earlobe. All stimuli were presented in a Ganzfeld dome. Dark-adapted responses from rods, combined rod and cone and oscillatory potentials followed by light-adapted photopic responses from single-flash cone and 30-Hz flicker were recorded. Signals were amplified, digitized, averaged and saved by a digital plotter (UTAS E-3000 System, LKC Technologies Inc., Gaithersburg, MD, USA). The peak-to-peak amplitude (μV) and the implicit time (ms) from each step of the ISCEV standard protocol were determined. The oscillatory potential amplitude was calculated as the sum of each wavelet and automatically analyzed by the UTAS E-3000 system (56). Amplitudes and peak times were classified in relation to normal values obtained in our own laboratory (57).

#### PhNR of the Light-Adapted ERG

Both pupils were dilated (pupil diameter >7 mm) with one drop each of tropicamide 1% and phenylephrine 10% and then light-adapted for 10 min followed by 1 min of preadaptation to the blue background light before the first stimulus. The corneal surface was anesthetized with two drops of tetracaine 1%. ERGs were registered with Dawson-Trick-Litzkow (DTL-Plus™) micro conductors (Diagnosys LLC, Lowell, MA, USA) attached to the nasal and temporal canthus with the fiber positioned on the lower border of the cornea. Gold cup electrodes were used in the temple for reference and Fz for ground. Electrode impedance was checked and set at 5 kilo Ohms (kΩ) or less. All stimuli were presented in a LED-based ColorBurst™ mini-ganzfeld handheld stimulator (Diagnosys LLC, Lowell, MA, USA) as previously described (58).

A flash of a red stimulus (640 nm) lasting 4 ms was recorded at a rate of 2 Hz against a blue (470 nm) background saturation. The flashing red stimulus was presented at 1 cd.s/m^2^, while the blue background remained at 10 cd/m^2^. Three sets of 50 sweeps lasting 150 ms were recorded using a bandpass filter between 0.3 and 300 Hz. The PhNR waveforms were amplified, digitized and saved by an Espion e2™ (Diagnosys LLC, Lowell, MA, USA). Each of the three repetitions was edited to eliminate artifacts and determine a constant average value. The records were obtained and analyzed from both eyes (58).

The a-wave, b-wave and PhNR were determined for each peak time (ms) and amplitudes (μV) by two experienced examiners (AB, GISB). PhNR was specified as a negative-going wave that occurs after the b-wave. PhNR can be measured from baseline to trough (BT, the amplitude to the trough of the PhNR measured from pre-stimulus baseline of 0 μV) or from peak to trough (PT, the amplitude between the peak of the b-wave and the trough of the PhNR). Wave ratios BT/b and PT/b were also evaluated (58).

### Fundus Photography and OCT Imaging

Dilated indirect ophthalmoscopy and fundus photography (iCam Camera, Optovue, Fremont, CA, USA) were performed. Spectral-domain OCT (iVue SD-OCT, Optovue, Fremont, CA, USA) was used for imaging of the macula and the optic nerve head from both eyes under pupil dilation. Automated segmentation (manually confirmed) and thickness analyses were performed for macular ganglion cell complex (GCC) and peripapillary retinal nerve fiber layer (RNFL) thickness. For peripapillary RNFL measurement, a 3.45 mm diameter circular scan centered on the optic disc was used and the data of four sectors (temporal, superior, nasal and inferior) were collected. Scans with a 6 × 6 mm circular field were used for acquire global macular GCC (comprised of retinal ganglion cell layer, inner plexiform layer and RNFL). The normal range of global macular GCC and RNFL thickness were considered from database of SD-OCT Optovue. The image acquisition software had its own quality indicator based on the signal power index (SQI), which classifies the mappings as “good” (if SQI ≥ 60) or “bad” (if SQI < 60). Images with segmentation failures, significant motion artifacts, or signal strength < 60 were excluded.

### Psychophysical Testing: Visual Acuity, Color Vision, and Visual Fields

Visual acuity was measured with current correction by using a retro-illuminated Early Treatment Diabetic Retinopathy Study (ETDRS) chart positioned at 4 m, expressed as a logarithm of the minimum angle of resolution (logMAR). Counting fingers, hand movements, light perception and no light perception, were respectively, converted to 1.8; 2.3; 2.8, and 3.0 logMAR (59).

Color discrimination was estimated by two distinct tests: The Pseudo-Isochromatic Plates for Testing Color Perception (American Optical Corporation) and the Farnsworth-Munsell 100 Hue Color Test (13). The Pseudo-Isochromatic Test is composed by 15 plates of numeric patterns of one or two digits and the score was set as the number of plates identified. Farnsworth-Munsell 100-hue color test was used for monocular color vision assessment and the scoring software was used to evaluate subject's color vision discrimination providing error score and axis.

Visual field testing was performed in either eye of affected and unaffected subjects according to the visual status. For eyes with VA <20/200 the Goldmann kinetic perimetry was performed whereas eyes with VA ≥20/200 had their visual fields tested with the Humphrey visual field analyzer (HVFA)with SITA-Standard 30-2 protocol (HFAII 750 Threshold Test, Carl Zeiss Meditec, Jena, Germany). Visual field defects were quantified by measurement of mean deviation (MD) in Humphrey visual field test and the occurrence of central scotoma (defined as isolated scotoma in the circular area between 0 and 10 degrees), cecocentral scotoma or full-field defect in both perimeters.

### Molecular Analysis

Genomic DNA was extracted from blood samples using the QIAamp DNA Mini Kit (Qiagen®). Screening of the m.11778G>A, m.3460G>A, and m.14484 T>C mutations was performed by Sanger Sequencing. We amplified the regions encompassing these mutations by polymerase chain reaction (PCR) with the following pairs of primers: (from mtDNA position 11,640 to 12,413) 5'- TAGCCCTCGTAGTAACAGCCATT-3' and 5'- GGGTTAACGAGGGTGGTAAGG-3'; (from mtDNA position 3,130 to 3,751) 5'- AGGACAAGAGAAATAAGGCC-3' and 5'- TGATGGCTAGGGGTGACTTCAT-3'; (from mtDNA position 14,150 to 14,810) 5' – CTATTCCCCGAGCAATCTCAATT-3' and 5' CCACATCATTCATCGACCTCC-3'. PCR products were purified with the QIAEX II Gel Extraction Kit (Qiagen®), and 90 ng of the purified product was used for sequencing reactions with BigDyeTM Terminator v3.1 Cycle Sequencing kit (Applied Biosystems TM), and according to manufacturer's instructions. Sequencing PCR products were electrophoresed in a 3,130 Genetic Analyzer (Applied Biosystems TM), and the obtained sequences compared to the revised Cambridge Reference Sequence (NC_12920).

The presence of heteroplasmy was confirmed by restriction fragment length polymorphism analysis (RFLP). For the m.11778G>A mutation, we amplified a region between mtDNA positions 11,640 and 12,898, which was digested with *Bms*I (*SfaN*I isoschizomer; Invitrogen^TM^). This fragment contains restriction sites for *Bms*I at positions 11,787, 12,466, and 12,813, giving rise to fragments with 147 bp, 679 bp, 347 bp, and 85 bp. The mutation abolishes the restriction site located at position 11787, generating fragments with 826 bp, 347 bp, and 85 bp. In the case of the m.3460G>A mutation, the fragment between positions 3,130 and 3,751 was digested with *Hin*1I (*Bsa*HI isoschizomer; Thermo Scientific). This fragment contains a restriction site for *Hin*1I, at position 3,459, generating two fragments: with 329 bp and 292 bp. The m.3460G>A mutation abolishes this restriction site. In both cases, digested products were run in a 2% agarose gel, stained with ethidium bromide, and visualized under UV light (60).

### Statistical Analysis

Statistical analyses were performed using Stata/SE Statistical Software, Release 14.0, 2015 (Stata Corp, College Station, Texas, USA). Frequency tables were used for descriptive analysis. The association of continuous results with categorical predictors was evaluated through Kruskal-Wallis test followed by *post-hoc* analysis of Dunn. Receiver operating characteristic (ROC) curve was constructed to determine the best cut-off of PhNR BT amplitudes for detecting affected participants as well as to determine sensitivity and specificity. Correlations between different continuous parameters were evaluated using Spearman correlation test. *P*-values ≤ 0.05 were considered statistically significant.

Both eyes from each participant were tested for all procedures. However, for statistical analysis, only data from one eye of each participant were included. In the affected group only data from the first affected eye were used whereas for carriers and controls only data from the right eye were used.

## Results

### Demographics, Genotype, Clinical Features, Color Vision, and Visual Field

A total of 41 individuals with suspected mutation of LHON were referred, from different geographical regions of Brazil (28 cities) and were genotyped for one of the three LHON pathogenic mtDNA mutations. Out of these, 38 (92.6%) participants (24 affected and 14 carriers of 19 families) had the confirmation of one of the three LHON pathogenic mtDNA mutations (m.11778G>A; m.14484T>C and m. 3460 G>A). In Table 1 demographics, genotype, clinical features (age at onset, visual acuity, interval between first and second affected eye and idebenone usage) for affected patients are shown. Demographics, genotype and visual acuity for unaffected carriers are shown in Table 2.

**Table 1:** Demographics, visual acuity, substance usage and idebenone therapy from 24 genotyped affected LHON participants.

**Subject**	**Family #**	**Age (years)**	**Sex**	**Genotype**	**VA (logMAR)**		**Age onset (years)**	**Interval between eyes (months)**	**Substance usage**	**Idebenone**
					RE	LE				
A1	1	14	M	m.11778G>A homoplasmic	1.9	**HM**	12	2	None	N
A2	2	16	M	m.11778G>A homoplasmic	1.2	**1.4**	11	0.5	None	N
A3	3^+^	17	M	m.11778G>A homoplasmic	0.2	**0.9**	16	0	None	Y
A4	4	19	M	m.11778G>A homoplasmic	**1.9**	1.9	15	1	None	Y
A5	3^+^	21	M	m.11778G>A homoplasmic	0.5	**CF**	20	0.5	None	N
A6	5	24	M	m.11778G>A homoplasmic	**1.3**	1.0	17	0.25	A	N
A7	6^+^	25	M	m.11778G>A homoplasmic	**0.7**	0	20	0	None	N
A8	6^+^	27	M	m.11778G>A homoplasmic	1.0	**1.2**	19	0	None	N
A9	7	27	M	m.11778G>A homoplasmic	1.6	**1.5**	25	4.5	A	N
A10	8	31	M	m.11778G>A homoplasmic	**HM**	HM	20	0	None	N
A11	9	33	M	m.11778G>A heteroplasmic	**1.5**	1.4	27	12	A	Y
A12	10	33	M	m.11778G>A homoplasmic	CF	**HM**	15	0	None	N
A13	11	33	M	m.11778G>A homoplasmic	**HM**	HM	25	5	None	N
A14	12^§^	39	F	m.11778G>A homoplasmic	CF	**CF**	11	0.25	None	N
A15	12^§^	62	M	m.11778G>A homoplasmic	**HM**	HM	18	6	A, T	N
		*$\bar{X}$ = 28.5 ± 27.4*					*$\bar{X}$ = 18.3 ± 4.8*	*$\bar{X}$ = 2.1 ± 3.4*		
A16	13	28	M	m.14484T>C homoplasmic	0.9	**1.0**	14	3	None	N
A17	14	39	M	m.14484T>C homoplasmic	**1.5**	1.0	38	5	A, T	N
A18	15	43	M	m.14484T>C homoplasmic	**1.4**	1.1	28	6	A	N
A19	16^+^	44	M	m.14484T>C homoplasmic	0.8	**0.9**	14	2	None	N
A20	16^+^	48	M	m.14484T>C homoplasmic	**0.4**	0.3	25	0.5	A, T	N
		*$\bar{X}$ = 40.9 ± 7.6*					*$\bar{X}$ = 24.1 ± 9.8*	*$\bar{X}$ = 3.2 ± 2.2*		
A21	17^*^	21	M	m.3460G>A heteroplasmic	**1.6**	1.8	14	0	A, T, C	N
A22	17^*^	21	M	m.3460G>A heteroplasmic	**1.8**	1.6	15	1	A, C	N
A23	18	25	M	m.3460G>A homoplasmic	**1.1**	1.3	22	2	None	Y
A24	19	31	M	m.3460G>A homoplasmic	1.8	**CF**	29	1	A, T	N
		*$\bar{X}$ = 24.7 ± 4.8*					*$\bar{X}$ = 20.5 ± 7.0*	*$\bar{X}$ = 1.0 ± 0.8*		
*Overall*		$\bar{X}$ *= 30.5 ± 11.4*					$\bar{X}$ *= 20.4 ± 6.4*	$\bar{X}$ *= 2.0 ± 2.9*		

* *index (A21) and twin brother*;

§ *index (A14) & maternal uncle*;

+ *index (A3, A20) and brother*.

*A, alcohol; C, Cannabis; CF, counting fingers; F, female; HM, hand movements; M, male; N, no; T, tobacco; Y, yes. Visual acuity from first affected eye is shown in bold; $\bar{X}$, mean value followed by ±standard deviation*.

**Table 2:** Demographics and visual acuity from 14 carrier LHON participants.

**Subject**	**Family #**	**Age (years)**	**Sex**	**Genotype**	**VA (logMAR)**	
					**RE**	**LE**
C1	12	14	F	m.11778G>A homoplasmic	0	0
C2	11	18	M	m.11778G>A heteroplasmic	−0.2	−0.2
C3	3	36	F	m.11778G>A homoplasmic	0	0
C4	12	36	F	m.11778G>A homoplasmic	0	0
C5	11	38	F	m.11778G>A heteroplasmic	−0.1	−0.1
C6	6	44	F	m.11778G>A homoplasmic	0.6	0
C7	6	45	F	m.11778G>A homoplasmic	0	1
C8	11	48	F	m.11778G>A heteroplasmic	0	0
C9	2	48	F	m.11778G>A homoplasmic	0	0
C10	11	56	F	m.11778G>A homoplasmic	0.1	0.2
C11	12	57	F	m.11778G>A homoplasmic	0.2	0.2
C12	13	58	F	m.14484T>C homoplasmic	−0.1	0
C13	18	47	F	m.3460G>A homoplasmic	0	0
C14	17	52	F	m.3460G>A heteroplasmic	0	0
		$\bar{X}$ = *43.2 ± 13.3*				

*F, female; M, male; logMAR, logarithm of the minimum angle of resolution; RE, right eye; LE, left eye; VA, visual acuity*.

*$\bar{X}$, mean followed by ± standard deviation*.

In the 24 LHON affected subjects (23 males, mean age = 30.5 ± 11.4 years; median: 28 years) the genotype was m.11778G>A [*N* = 15 (62.5%)]; m.14484T>C [*N* = 5 (20.8%)] and m.3460G>A [*N* = 4 (16.7%)]. Carriers (mean age: 43.2 ± 13.3 years) were 13 females and one male [m.11778G>A – *N* = 12 (78.5%), m.14484T>C – *N* = 1 (7.2%), and m.3460G>A – *N* = 2 (14.3%)]. Controls were eight females and seven males (mean age: 32.6 ± 11.5 years).

The age onset vision loss ranged from 11.8 to 38.0 (mean 20.4 ± 6.4 years; median: 19.8). The duration of symptoms ranged from 5 to 516 months (mean 120 ± 129.6 months; median: 78.4 months) and the visual acuity was severely impaired in both eyes in most cases (mean logMAR 1.65 ± 0.90; median: 1.5). The right eye was first affected in 54% of subjects, with 4 (16%) affected subjects on continuous use of idebenone.

Color discrimination tests were performed in 22 affected, with two affected not able to be tested due to severe vision loss (subjects A15 and A24). All tested affected subjects showed severe diffuse dyschromatopsia. All asymptomatic carriers had normal Pseudo-Isochromatic test scores in both eyes, with 10 showing low color discrimination scores in at least one eye in Farnsworth-Munsell 100 Hue Color Test (error scores ranging from 40 to 400 losses were detected in 10/14 LHON asymptomatic carriers).

Ten affected subjects had both eyes tested with HVFA, with reliable results in five subjects (A3, A7, A16, A19, and A20); in two subjects (A3 and A16) absolute central scotoma were found in both eyes; cecocentral scotoma in one eye and central scotoma in the contralateral eye were found in two brothers with m.m.3460G>AT>C mutation (A19 and A20) and in one subject (A7) there was a central scotoma in the right eye and normal exam in the left eye. In 13 affected subjects the visual fields were tested by Goldmann perimetry, with 11 of them showing absolute central scotoma in both eyes (A1, A2, A4, A8, A9, A12, A13, A14, A21, A22, and A23), one subject with only small peripheric island of vision in both eyes (A15) and one subject (A10) with a peripheric island of vision in one eye and central scotoma in the contralateral eye (A10). In one subject (A17) HVF disclosing ceco-central scotoma in left eye and central scotoma in his right eye by Goldmann perimetry.

All 14 carriers had both eyes tested by HVFA with reliable results in 12 subjects. In 10 subjects the visual fields were completely normal in both eyes (C1, C2, C3, C4, C5, C8, C9, C11, C12, and C13). Central scotoma in the right eye and normal VF was found in one subject (C6) and normal VF in the right eye with central scotoma in the left eye (C7).

### Photopic Negative Response

**Figure 1:**
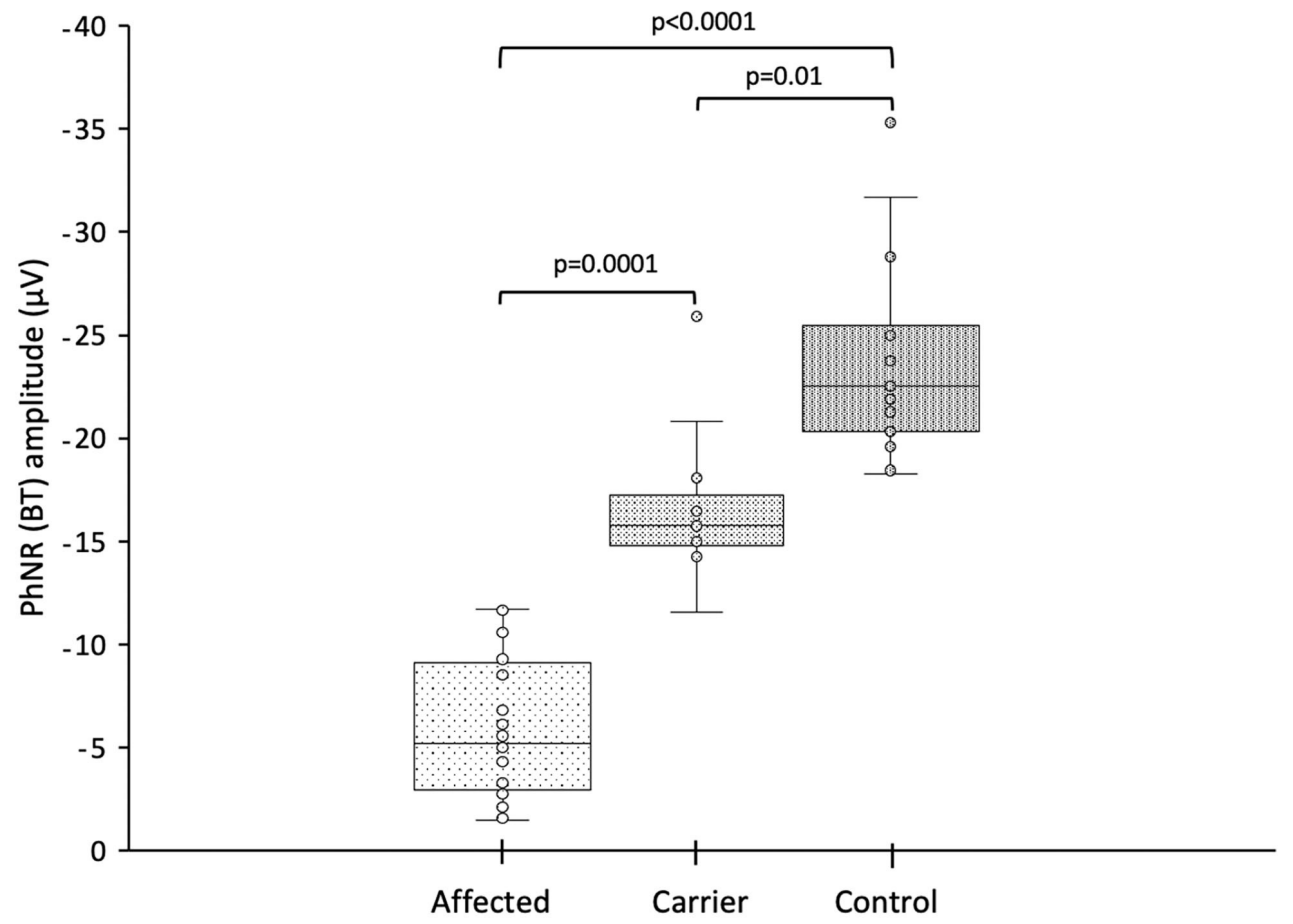
Photopic negative response amplitude (BT) from LHON affected patients, LHON asymptomatic carriers and controls (each box shows the median, quartiles, and extreme values; circles represent the subjects).

The PhNR parameters (a-wave, b-wave, BT, PT, BT/b and PT/b) for the LHON affected, LHON carrier and control subjects are summarized and compared in Table 3 and shown in Figure 1. PhNR (BT, BT/b and PT/b) amplitudes were significantly reduced (*p* < 0.0001) in LHON affected (BT = −5.96 ± 3.37 μV) compared to carriers (BT = −16.53 ± 3.4 μV) and controls (BT = −23.91 ± 4.83 μV), and in carriers compared to controls (*p* < 0.0001). PhNR amplitudes either by BT or PT were comparable among the three LHON mtDNA pathogenic mutations. There was no correlation between PhNR amplitude (BT) with age, use of idebenone or duration of symptoms.

**Table 3:** Mean, median and respective standard deviations PhNR amplitudes (BT and PT), PhNR amplitude ratios (PT/b and BT/b) and PhNR peak times of affected, carrier and controls.

	**Affected**	**Carrier**	**Control**	**p-value^Ɨ^**
	**Mean ± SD (median)**	**Mean ± SD (median)**	**Mean ± SD (median)**	
Amplitude (μV)
PhNR (BT)	−5.96 ± 3.37 (5.20)^*^	−16.53 ± 3.41 (15.78)^**^	−23.91 ± 4.83 (22.54)	**0.0001**
PhNR (PT)	−105.53 ± 28.43 (105.98)^*^	−138.27 ± 35.63 (127.92)	−121.64 ± 30.27 (117.07)	**0.0002**
PhNR Amplitudes ratios
BT/B (μV)	0.06 ± 0.04 (0.06)^*^	0.14 ± 0.02 (0.14)^**^	0.27 ± 0.11 (0.24)	**0.0001**
PT/B (μV)	1.06 ± 0.04 (1.06)^*^	1.14 ± 0.02 (1.14)^**^	1.27 ± 0.11 (1.24)	**0.0001**
Peak times (ms)
PhNR	62.54 ± 2.03 (60.07)	63.58 ± 2.95 (63.63)	62.79 ± 3.38 (62.98)	0.6677

Ɨ *Kruskal-Wallis test*.

*BT, baseline to trough; μV, microvolts, ms, milliseconds; PhNR, photopic negative response; PT, peak-to-trough; SD, standard deviation*.

* *p < 0.05 by Dunn test, comparing affected with carrier and affected with control*.

** *p < 0.05 by Dunn test, comparing carrier with control*.

**Figure 2:**
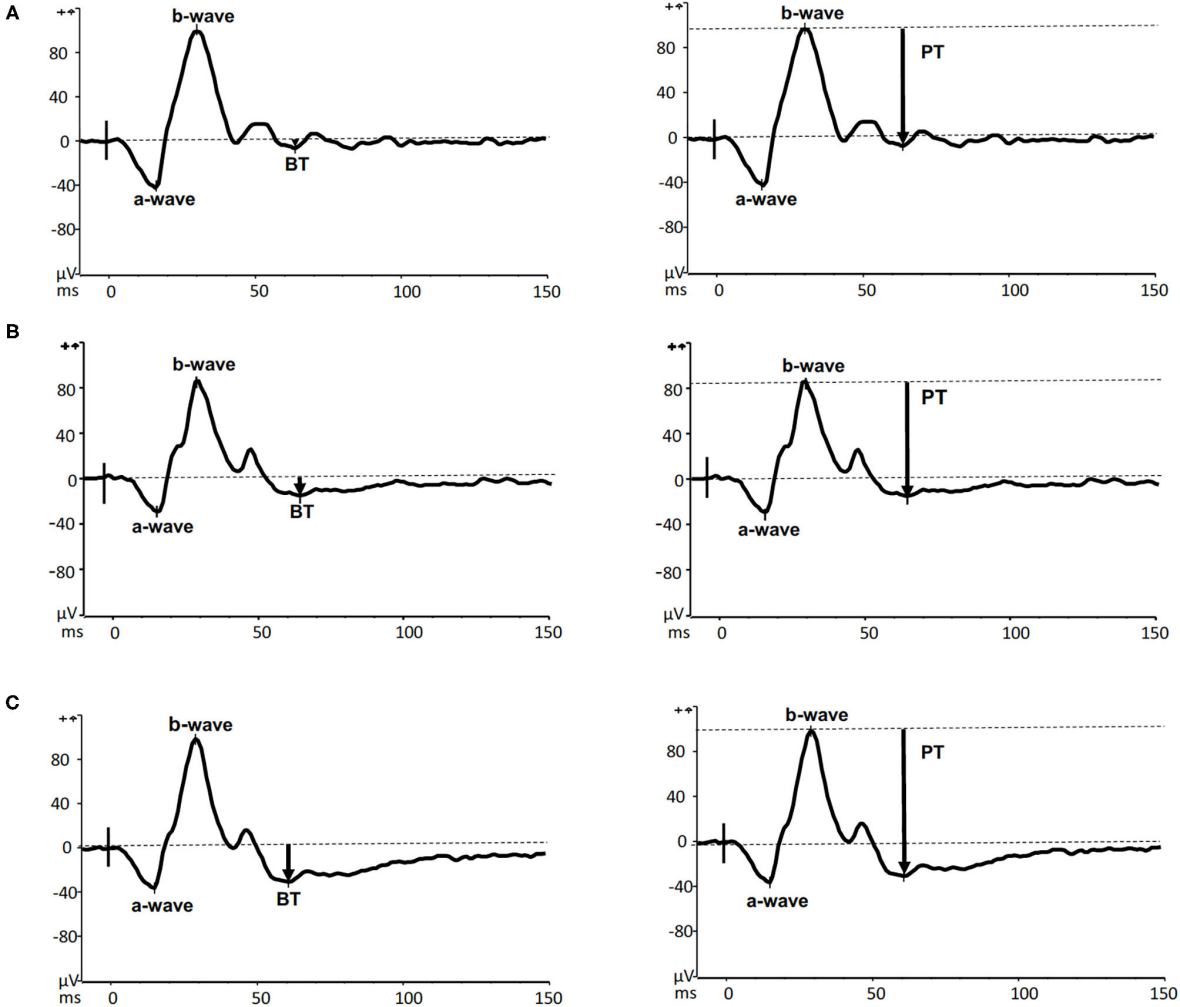
Representative PhNR recordings with amplitudes measured by both BT (left panels) and PT (right panels) from an m.11778G>A LHON affected (A13–upper panels), his unaffected carrier sister (C 5–middle panels) and a healthy 41-year-old male control (lower panels).

**Figure 3:**
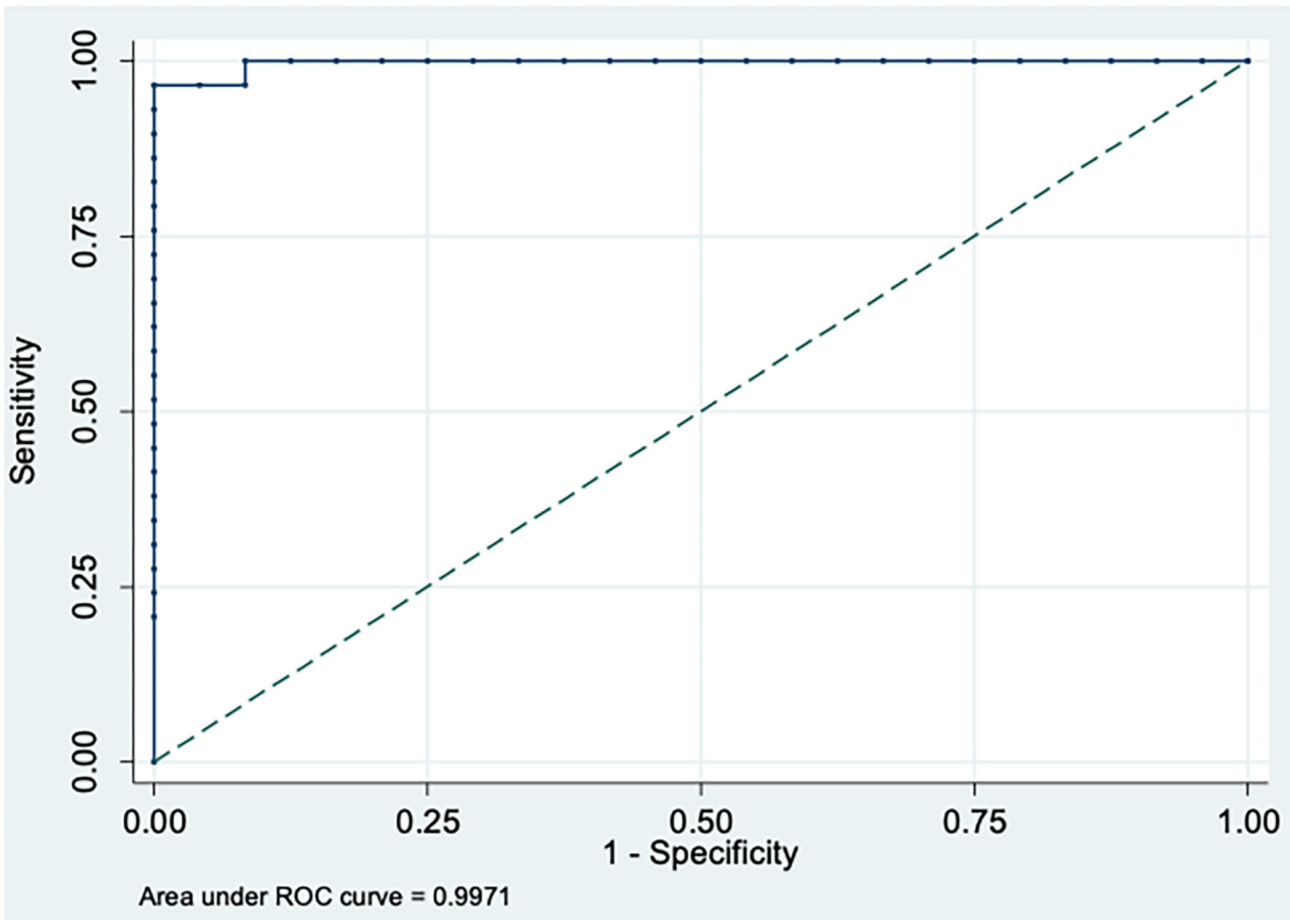
Receiver Operating Characteristic (ROC) curve for affected vs. controls plotted at best cut-off at 11.72 μV for PhNR amplitude (BT) showing high sensitivity and specificity with area under the curve (AUC) = 0.997 (95%CI: 0.990–1.000).

**Figure 4:**
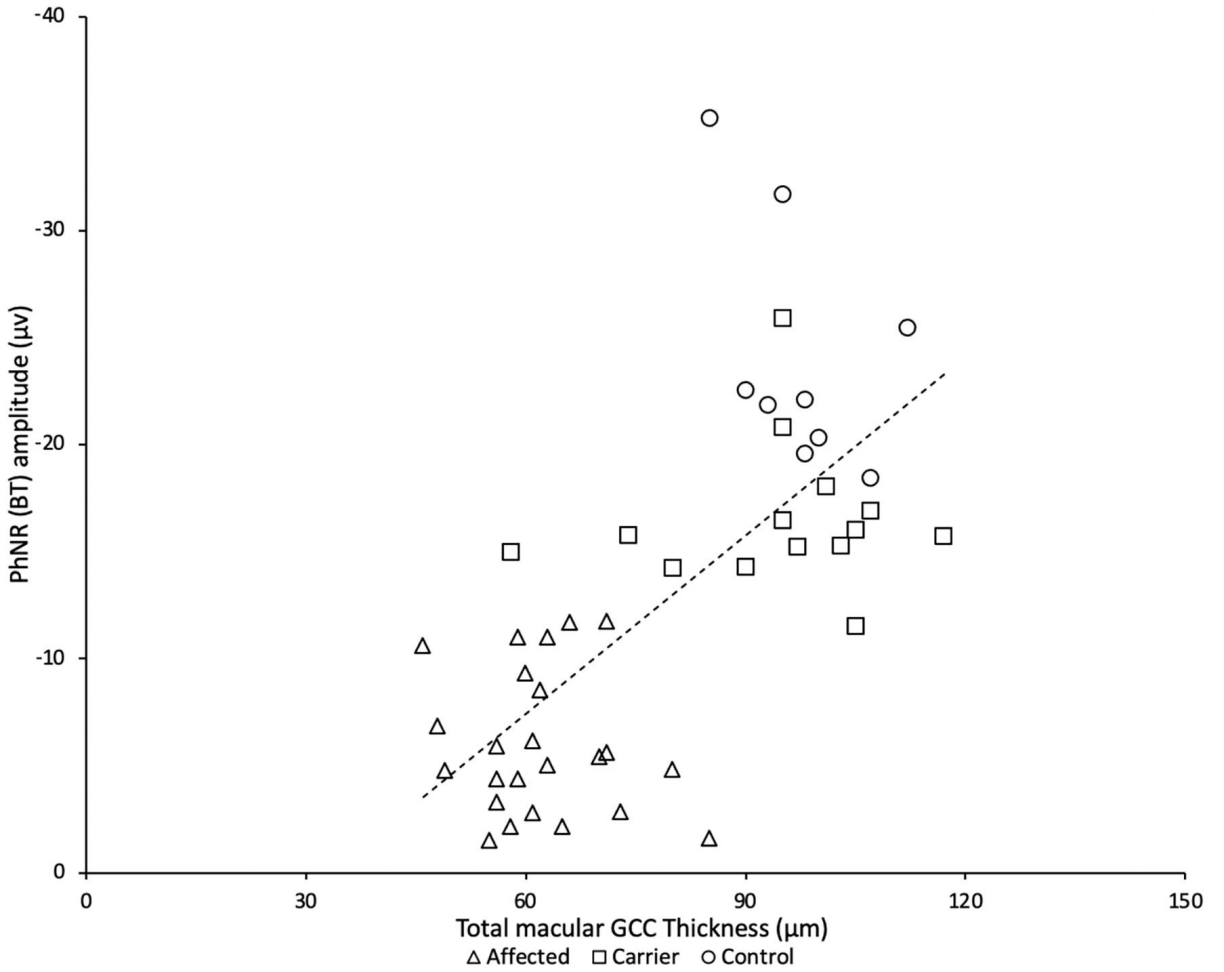
There was a significant correlation between PhNR (BT) and total macular ganglion cell complex (GCC) from LHON affected patients (*N* = 24), asymptomatic carriers (*N* = 14) and controls (*N* = 8).

Representative PhNR amplitudes for affected, carrier and control individuals are shown in Figure 2. ROC curve analysis revealed PhNR amplitude of BT to be a good parameter (Figure 3) to detect cases yielding a positive predictive value of 100%, a sensitivity of 96.5% and specificity of 100% at the cutoff of 11.72 μV. PhNR amplitude (BT) was significantly correlated (*r* = −0.62; *p* < 0.05) with the macular GCC thickness in affected, carrier and control as shown in Figure 4.

### Fundoscopy and OCT Imaging

Bilateral optic atrophy was found LHON affected subjects, except subject A5 who presented in the acute phase of the disease with optic disk edema and peripapillary telangiectasia in his right eye and mild temporal optic disk pallor in his left eye.

**Figure 5:**
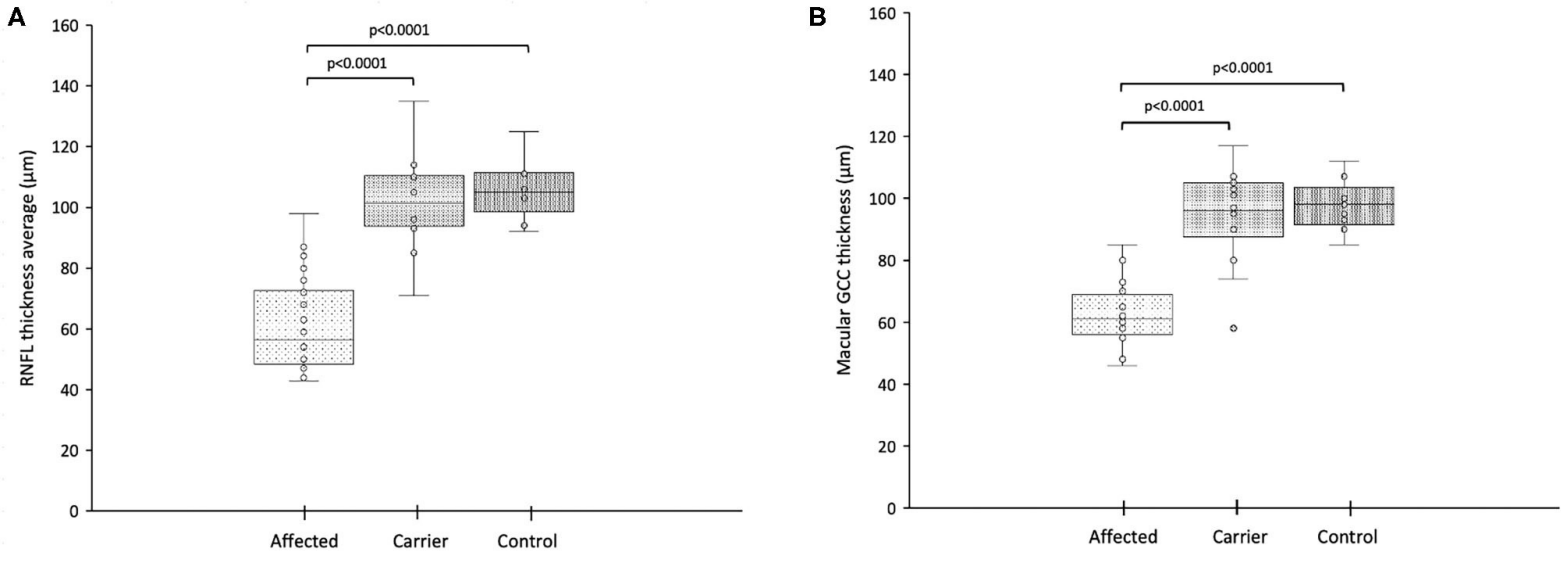
**(A)** Retinal nerve fiber layer thickness in the 360-degree average from LHON affected patients, LHON asymptomatic carriers and controls (each box shows the median, quartiles, and extreme values; circles represent individual subjects); **(B)** Total macular ganglion cell complex thickness from LHON affected (each box shows the median, quartiles, and extreme values; circles represent individual subjects).

**Figure 6:**
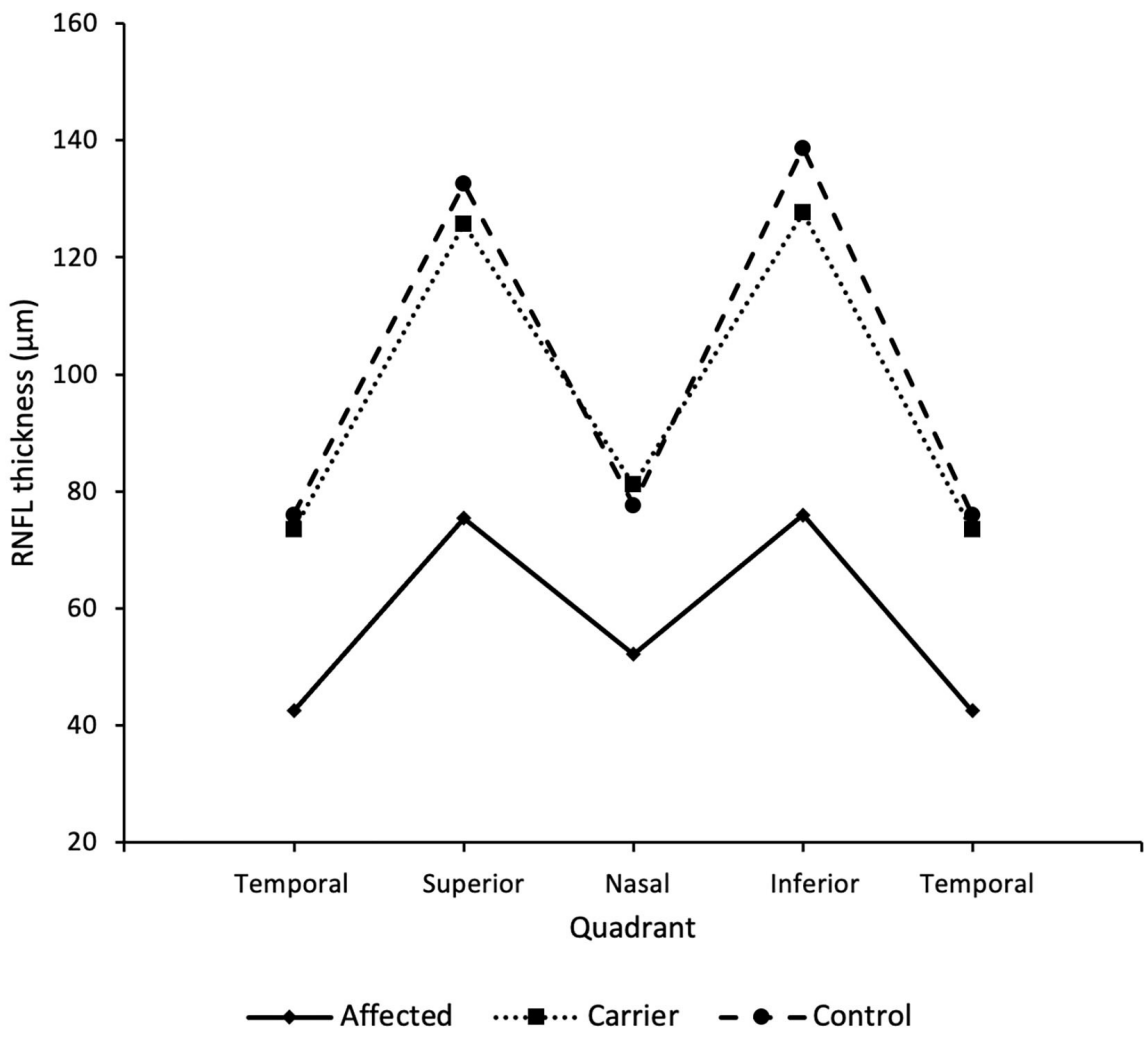
RNFL thickness in each quadrant of LHON affected, LHON asymptomatic carriers and controls, showing a significantly thinner RNFL in all quadrants for LHON affected compared to carriers and controls (*p* < 0.0001).

**Figure 7:**
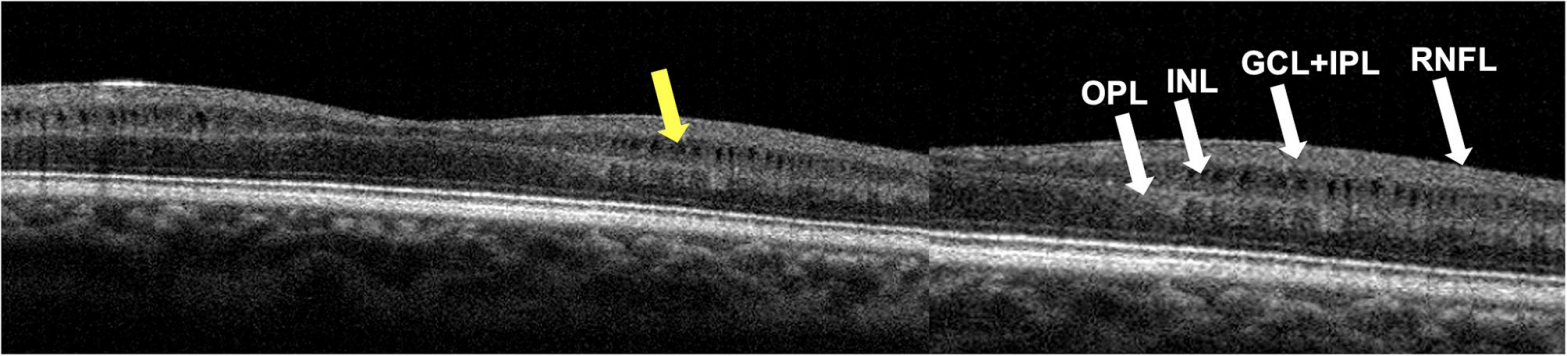
Foveal OCT scan from subject A8's right eye. Segmentation identifies macular microcysts of inner nuclear layer (INL) as pointed by the yellow arrow. Retinal nerve fiber layer (RNFL), ganglion cell layer (GCL), inner plexiform layer (IPL) and outer plexiform layer (OPL) are shown on the right panel.

Macular SD-OCT revealed selective loss of the global macular GCC thickness in affected LHON compared with carrier and control as shown in Table 4 and Figure 5 (*p* < 0.001). Global macular GCC thickness did not show significant changes in unaffected carrier compared to control. This occurred in parallel with loss of the average peripapillary RNFL thickness and was similar in temporal, nasal, inferior, and superior quadrants (*p* < 0.001) (Figure 6). In one particular family (#6) macular microcysts were found (Figure 7). The unaffected mother (C7) had strabismic amblyopia in her left eye (VA 20/200) and 20/20 vision in her right eye, with few microcysts in the macular innermost retina in the left eye. Her two affected sons (A7 and A8) had macular microcysts, A7 in right eye and A8 in both eyes.

**Table 4:** Peripapillary retinal nerve fiber layer thickness and macular ganglion cell complex, as measured by optical coherence tomography.

	**Affected**	**Carrier**	**Control**	***p*-value^Ɨ^**
	**Mean ± sd (median)**	**Mean ± sd (median)**	**Mean ± sd (median)**	
RFNL thickness (μm)
Average	61.58 ± 15.24 (56.50)^*^	101.86 ± 15.08 (101.50)	105.67 ± 9.87 (105.00)	0.0001
Superior	75.42 ± 25.19 (71.00)^*^	125.79 ± 21.51 (128.00)	132.56 ± 20.12 (128.00)	0.0001
Temporal	42.46 ± 9.62 (41.00)^*^	73.43 ± 16.07 (75.50)	76.00 ± 6.56 (77.00)	0.0001
Inferior	76.08 ± 18.42 (74.00)^*^	127.64 ± 21.71 (132.00)	138.78 ± 14.45 (139.00)	0.0001
Nasal	52.21 ± 15.88 (49.50)^*^	81.14 ± 9.28 (80.00)	77.67 ± 8.73 (79.00)	0.0001
Macular GCC thickness (μm)	62.21 ± 9.43 (61.00)^*^	94.43 ± 15.16 (96.00)	97.56 ± 8.26 (98.00)	0.0001

Ɨ *Kruskal-Wallis test*.

*RFNL, retinal nerve fiber layer; GCC, macular ganglion cell complex; μm, micra*.

* *p < 0.05 by Dunn test, comparing affected with carrier and affected with control*.

### PRVEP, FVEP, and ff-ERG

Abnormalities in PRVEPs were found in all affected individuals, with 12 (50%) showing undetectable PRVEP for both 15' and 60'check sizes (A1, A4, A9, A10, A11, A12, A13, A14, A15, A21, A22, and A24). In 4 affected patients (17%) there were abnormal responses (reduced amplitudes and delayed latencies) for both check sizes in both eyes (A3, A8, A16, and A20). Non-detectable responses only for the smaller checks along with abnormal responses for larger checks in both eyes were found in 3 (13%) affected (A6, A19, and A23). Responses for smaller checks with either reduced amplitude or delayed latencies in at least one eye were found in 4 (17%) affected (A2, A5, A17, and A7). One patient (A10) showed undetectable responses for both check sizes in one eye and abnormal response for the larger checks in the contralateral eye.

For those 12 affected patients with undetectable pattern-reversal responses in both eyes for both check sizes, normal flash VEPs in both eyes were found in seven (A4, A9, A12, A13, A15, A21, and A22) whereas abnormal responses in both eyes were found in the remaining (A1, A10, A11, A14, and A24). In 12 patients with PRVEP recordable responses, FVEP normal responses for both eyes were found in 6 participants (A7, A16, A17, A19, A20, and A23), two patients showed normal response in one eye and delayed latencies in the contralateral eye (A3, A5) and in three patients (A6, A8, and A18) abnormal flash VEPs were found in both eyes.

All carriers had normal pattern-reversal and flash VEPs in both eyes, except two participants with previous strabismic amblyopia (C6 and C7) disclosing only abnormalities (reduced PRVEP P100 amplitudes) in their amblyopic eyes.

Normal scotopic and photopic ff-ERGs in both eyes (ISCEV standard protocol) were found in 38 participants with two affected (A7 and A8) showing reduced b/a ratio for the maximal response in both eyes consistent with OCT findings of macular microcysts in both eyes.

## Discussion

The assessment of the ganglion cell function by PhNR in LHON carrier and affected subjects confirmed and extended previous findings with significantly reduced mean PhNR amplitude (BT) and the PhNR/bwave amplitudes ratios (BT/b and PT/b) in both affected and carriers compared to the responses from the normal controls (32, 53). Accordingly, the ROC analysis also confirmed that the PhNR (BT) amplitude showed the best discrimination between control, LHON carrier and affected groups confirming findings from the SOA-BR pedigree (32). The current findings suggest that the PhNR amplitude can reveal functional abnormality in LHON carriers with normal vision while the SD-OCT decreases later in the course of the chronic disease in affected subjects. Our study also indicated severe RGC dysfunction in LHON affected subjects. The amplitude of PhNR in LHON does not seem to be influenced by the specific mtDNA mutation, visual acuity, age or duration of symptoms.

Our results demonstrate the usefulness of the PhNR in both clinical care and research of diseases affecting RGCs, as LHON. Since the PhNR objectively and quantitatively reflects the overall function of the RGCs, it seemed a suitable quantitative test to monitor disease severity and could also represent a useful additional tool in clinical trials to investigate new therapeutic approaches for these conditions. This can be confirmed by the significant correlation found between PhNR amplitude and macular GCC thickness assessed by OCT. Furthermore, the PhNR offers advantages over other electrophysiological tests based on pattern stimuli, since it is a full field test that does not require clear optics or refractive correction. To note, PhNR, as other electrophysiological tests, might precede structural changes or even monitor changes at a different rate than changes in visual structure and function.

The affected LHON subjects showed a significant reduction in macular GCC and RNFL thickness in all quadrants compared with carriers and control subjects. The present study provides key corroboration with previous investigations that reveal OCT an important feature in structural analyses of LHON including optic disk size, RNFL and GCC (22, 23, 61). Furthermore, it has been showed that RGCs loss occurs before RNFL in time of LHON visual loss in acute vision loss, whereas in our study mostly chronic LHON affected subjects were included and we found diffuse reduction of RGCs and RFNL thickness (34). A statistically significant association between the PhNR BT amplitude and total macular ganglion cell complex thickness using SD-OCT was found. Since PhNR is likely to reflect the activity of RGCs, this linear relationship between function and structure was already described in previous studies and indicates that RGCs function declines proportionately with neural loss (32).

In Family#6 (homoplasmic m.11778G>A), macular microcysts were found in a carrier (C7) and her two sons (A7 and A8), consistent with previous findings including the SOA-BR pedigree which demonstrate that macular microcysts occur in about 5.6% of patients with LHON (29). Some authors have proposed that the young age of these patients supports the hypothesis of vitreoretinal adhesion and traction, as vitreous is known to be more firmly adherent in youth (62). It has also been proposed that macular microcysts are caused by the effects of trans-synaptic retrograde degeneration (63, 64).

### Other Tests and Demographics

Since currently mtDNA testing is not available in the Brazilian public health system (Sistema Único de Saúde – SUS), the opportunity to have genotype provided free-of-charge, as part of this study, was of assistance to patients. Out of the 24 affected subjects, 12 (A1, A2, A3, A4, A5, A6, A7, A8, A9, A12, A17, and A24) were from very low-income sociodemographic background and anxious for the diagnosis confirmation, with a waiting time up to 18 years (mean = 4.5 years). Limitations of this study were the genotype testing including only the three more frequently found mtDNA mutations, which excluded three recruited individuals that might harbor other mutations and the restrict recruitment interval that might have impacted the sample size.

In this group of 19 Brazilian families genotyped for LHON the distribution of the three most common mtDNA mutations found was comparable to previous reports from other parts of the world with 62% of m.11778G>A, 21% of m.14484T>C and 16% of m.3460G>A (6, 7). While it is known that LHON affects prevalently males and in the studied sample there was only one female (4%) affected at 11 years of age among the 24 affected subjects compared to 15% affected females in the original description of the SOA-BR pedigree (8). We believe that this male:female ratio is an underestimation related to the recruitment interval and the small sample size. Longitudinal studies with larger samples could provide a better representation of the disease in Brazil.

An international consensus has recommended idebenone as the standard therapy for LHON, mainly in the acute phase of the disease (65). However, this substance is not registered in the Brazilian regulatory agency (Agência Nacional de Vigilância Sanitária – ANVISA) as a therapy for LHON and consequently is not available in the market. In our sample only 17% of affected subjects reported idebenone treatment, with some of them obtaining the medication after legal appeal. To note, one patient who was not under idebenone therapy (A7 m.11778G>A) referred spontaneous recovery of vision in his left eye, with 20/20 visual acuity, relative central scotoma and reduced responses only for the smaller check size in the PRVEP. This particular case points out that recovery might have implications in therapeutic approaches (66).

A pair of monozygotic twin brothers harboring the m.3460G>A mutation presented the disease onset quite closely from each other (Table 1 subjects A21 and A22). Subject A21 developed visual loss at the age of 14 years in his right eye being affected firstly followed by left eye less than a month later, whereas the twin brother (A22) had visual loss at 15 years of age also in his right followed by left eye a month later. This concordant LHON cases in monozygotic twin brothers had already being reported with m.11778G>A (67) and m.14484T>C (68) mutations with the patients showing similar genotypic and clinical features as the pair of Brazilian twin brothers.

Our study shows the severe abnormalities in psychophysical and electrophysiological tests found in affected subjects as diffuse dyschromatopsia, central scotoma and reduced or non-recordable PRVEPs. A number of studies have reported abnormal PRVEP and flash VEP responses in LHON affected subjects (11, 69). Subclinical abnormalities in PhNR, color discrimination and PRVEPs were present in some carriers, confirming findings from the SOA-BR pedigree (12, 13, 21, 32).

In this representative cohort of Brazilian families with LHON the impairment of the ganglion cell function assessed by photopic negative response was found in both affected and carrier subjects harboring one of the three most frequent pathogenic mutations. These results show that PhNR is a promising tool for future clinical trials and function-structure studies in this disease. The present study provided important demographic features of LHON in Brazilian families as the distribution of the three major mtDNA mutations and gender prevalence along with the clinical and electrophysiological characterization of affected and carrier individuals.

## Data Availability Statement

The raw data supporting the conclusions of this article will be made available by the authors, without undue reservation.

## Ethics Statement

The studies involving human participants were reviewed and approved by the Committee of Ethics in Research of Federal University of São Paulo. All procedures performed in studies involving human participants were in accordance with the ethical standards of the institutional and/or national research committee and with the 1964 Helsinki declaration, its later amendments or comparable ethical standards. All participants provided informed consent. (Reference of ethics committee approval: UNIFESP-Hospital São Paulo - CAAE: 89695718.0.0000.55.05). Written informed consent to participate in this study was provided by the participants' legal guardian/next of kin.

## Author Contributions

AB, SS, CT, RK, VC, RB, and AS: conceptualization. AB, GB, PSa, DR, PSi, FM, SS, and SW: data collection. AB, GB, SS, CT, and AF: data analysis. AB, GB, SS, CT, RK, FM, DR, PSi, AF, SW, PSa, RB, VC, and AS: writing and review of manuscript. AB, SS, RB, RK, VC, and AS: funding acquisition. All authors contributed to the article and approved the submitted version.

## Conflict of Interest

The authors declare that the research was conducted in the absence of any commercial or financial relationships that could be construed as a potential conflict of interest.

## Abbreviations

BT: Baseline to trough
SOA-BR: Brazilian pedigree with m.11778G>A/haplogroup J LHON
DTL-Plus™: Dawson-Trick-Litzkow
ETDRS: Early Treatment Diabetic Retinopathy Study
UNIFESP: Federal University of São Paulo
N75: First negative deflection
P100: First positive deflection
FVEP: Flash Visually Evoked Potential
ff-ERG: Full-field electroretinogram
GCC: Ganglion cell complex
GCL: Ganglion cell layer
IPL: Inner plexiform layer
ISCEV: International Society of Clinical Electrophysiology of Vision
kΩ: Kilo Ohms
LHON: Leber's hereditary optic neuropathy
logMAR: Logarithm of the minimum angle of resolution
MD: Mean deviation
μV: Microvolt
ms: Milisecond
mtDNA: Mitochondrial DNA
nm: Nanometer
OPL: Outer plexiform layer
PRVEP: Pattern-Reversal Visually Evoked Potential
PT: Peak to trough
PhNR: Photopic negative response
ROC: Receiver operating characteristic
RGCs: Retinal ganglion cells.
